# Loss to Follow-Up as a Competing Risk in an Observational Study of HIV-1 Incidence

**DOI:** 10.1371/journal.pone.0059480

**Published:** 2013-03-12

**Authors:** Susan M. Graham, Janet Raboud, R. Scott McClelland, Walter Jaoko, Jeckoniah Ndinya-Achola, Kishor Mandaliya, Julie Overbaugh, Ahmed M. Bayoumi

**Affiliations:** 1 Departments of Medicine and Global Health, University of Washington, Seattle, Washington, United States of America; 2 Department of Medical Microbiology, University of Nairobi, Nairobi, Kenya; 3 Dalla Lana School of Public Health, University of Toronto, Toronto, Ontario, Canada; 4 Division of Clinical Decision Making and Health Care, University Health Network, Toronto, Ontario, Canada; 5 Department of Epidemiology, University of Washington, Seattle, Washington, United States of America; 6 PathCare, Mombasa, Kenya; 7 Human Biology and Public Health Sciences Divisions, Fred Hutchinson Cancer Research Center, Seattle, Washington, United States of America; 8 Centre for Research on Inner City Health, Keenan Research Centre of the Li Ka Shing Knowledge Institute and Division of General Internal Medicine, St. Michael's Hospital, Toronto, Ontario, Canada; 9 Department of Medicine and Institute of Health Policy, Management, and Evaluation, University of Toronto, Toronto, Ontario, Canada; University of Texas Medical Branch, United States of America

## Abstract

**Objective:**

Conventional survival estimates may be biased if loss to follow-up (LTF) is associated with the outcome of interest. Our goal was to assess whether the association between sexual risk behavior and HIV-1 acquisition changed after accounting for LTF with competing risks regression.

**Methods:**

HIV-1-seronegative women who enrolled in a Kenyan sex worker cohort from 1993–2007 were followed prospectively and tested for HIV at monthly clinic visits. Our primary predictor was self-reported sexual risk behavior in the past week, analyzed as a time-dependent covariate. Outcomes included HIV-1 acquisition and LTF. We analyzed the data using Cox proportional hazards regression and competing risks regression, in which LTF was treated as a competing event.

**Results:**

A total of 1,513 women contributed 4,150 person-years (py), during which 198 (13.1%) acquired HIV-1 infection (incidence, 4.5 per 100 py) and 969 (64.0%) were LTF (incidence, 23.4 per 100 py). After adjusting for potential confounders, women reporting unprotected sex with multiple partners were less likely to be lost to follow-up (adjusted sub-hazard ratio (aSHR) 0.50, 95% confidence interval (CI) 0.32–0.76, relative to no sexual activity). The risk of HIV-1 acquisition after reporting unprotected sex with multiple partners was similar with Cox regression (adjusted hazard ratio (aHR) 2.41, 95% CI 1.36–4.27) and competing risks regression (aSHR 2.47, 95% CI 1.33–4.58).

**Conclusions:**

Unprotected sex with multiple partners was associated with higher HIV-1 acquisition risk, but lower attrition. This differential attrition did not substantially bias Cox regression estimates when compared to competing risks regression results.

## Introduction

HIV-1 acquisition is often studied within the context of observational cohort studies, which typically recruit higher risk populations such as HIV-1-seronegative female sex workers (FSW) and follow them at regularly scheduled visits [1], [2]. Although such studies are valuable, they are susceptible to biases relating to the selection and retention of participants. The motivation to participate in research is influenced by the perceived risk of HIV-1 acquisition as well as social, economic, and political factors such as access to care, poverty, migration, stigma, and criminalization of sex work [3]. Because participants may drop out of cohort studies, statistical methods have been developed to impute missing data or weight data based on the duration of participation. Unfortunately, these methods do not adequately account for attrition bias, the differential (non-random) attrition among participant subgroups [4].

We studied attrition and HIV-1 acquisition risk in an established FSW cohort based in Mombasa, Kenya. Women who participated were initially HIV-1-seronegative and healthy. Although detailed reasons for loss to follow-up (LTF) were not collected, anecdotal reports suggest that common reasons included relocating and stopping sex work for a new job or steady partner. Thus, LTF could be associated with a lower HIV-1 risk. In contrast, Kaplan-Meier survival curves assume non-informative censoring – that is, they assume that individuals who were LTF were at the same risk of acquiring HIV-1 as those who continued to be followed. The aims of this study were to answer the following questions in the Mombasa FSW cohort:

1. Is sexual risk behavior associated with cohort attrition?
2. If cohort attrition is differential, how does this affect estimated associations between sexual risk behavior and HIV-1 acquisition?

## Methods

### Population

All HIV-1-seronegative women who enrolled in the Mombasa FSW Cohort between 1993 and 2007 and had at least one follow-up visit were eligible to be included in this analysis. Participants either experienced one of the two outcomes (i.e., HIV-1 acquisition or LTF) during the study period or underwent administrative censoring on December 31, 2007. We excluded visits that occurred in the context of two small randomized controlled trials (RCT) [5], [6], because trial participation might be associated with different rates of LTF or HIV-1 acquisition than routine care. Visits that occurred prior to RCT participation were included. RCT participants rejoined the cohort after conclusion of the trial or were censored at RCT enrolment if HIV-1 acquisition or LTF occurred during trial participation.

### Clinic procedures

Detailed study procedures have been described previously [7], [8]. Briefly, at the enrollment visit, data on demographic characteristics, sexual behavior, and medical history were collected by an interviewer using a standardized questionnaire. Women then underwent a standardized physical examination, including a pelvic speculum examination with screening for sexually transmitted infections (STI). At monthly follow-up visits, participants were asked about interim sexual behavior and medical history, underwent a standardized physical examination, and were screened for STI and HIV infection. All women received risk-reduction counseling and free condoms. Women with an STI received appropriate treatment.

All participants provided written informed consent. The cohort study was approved by the ethical review committees of the University of Nairobi and the University of Washington; this analysis was also approved by the University of Toronto Office of Research Ethics.

### Laboratory procedures

Screening for HIV-1 was performed using an ELISA (Detect-HIV; Biochem Immunosystems), and positive results were confirmed by a second ELISA (Recombigen; Cambridge Biotech). Serologic testing for HSV-2 was performed using a type-specific, HSV-2 gG-based ELISA (HerpeSelect; Focus Diagnostics, Cypress, California, USA). Samples with index values >1.1 were defined as seropositive, according to the manufacturer's instructions and previous analysis in this cohort [9].

Light microscopy of vaginal wet preparations at 40× magnification was used to diagnose vaginal trichomoniasis and candidiasis on the basis of identifying motile trichomonads and yeast forms, respectively. Bacterial vaginosis was evaluated by microscopy of vaginal Gram stains [10]. The number of polymorphonuclear leukocytes (PMNL) in three non-adjacent high power fields on microscopy of cervical Gram stains was quantified. Cervicitis was defined as the presence of ≥30 PMNL/high-power field of Gram-stained cervical secretions.

### Outcome definitions

HIV-1 acquisition was defined according to established procedures in the Mombasa Cohort [11]. Briefly, when HIV-1 RNA was detected in a plasma sample collected before seroconversion, the date of HIV-1 acquisition was estimated at 17 days before the first RNA-positive sample. For women without detectable HIV-1 RNA before seroconversion or with no pre-seroconversion samples available for RNA testing, the date of HIV-1 acquisition was estimated at the midpoint between the last seronegative and first seropositive clinic visits.

Loss to follow-up was defined as a last visit 90 days or more before the administrative censoring date. This definition was developed following a method described by Chi et al. to minimize misclassification bias [12]. We evaluated thresholds for defining LTF by calculating sensitivity, specificity, and misclassification rates based on visits that occurred during 2008 (i.e., the year after follow-up for this analysis ended). We established the LTF threshold at the cut point that minimized misclassification of both false positives and false negatives combined.

### Predictors and potential confounders

The primary predictors were reported partner numbers and condom use in the week before each study visit; these parameters were closely associated in distinct patterns. Specifically, during a given reporting week, women reporting no partner had no condom use (by definition), women reporting one partner usually reported always or never using condoms, and women reporting multiple partners used condoms more consistently than those reporting only one partner. Therefore, we combined these variables into a primary predictor variable that classified women according to their reported sexual risk behavior during a reporting week into five categories:

1. No sexual activity (reference group),
2. Only one partner with 100% condom use (referred to as “one partner, protected”),
3. Only one partner with <100% condom use (referred to as “one partner, unprotected”),
4. Multiple partners with 100% condom use (referred to as “multiple partners, protected”), and
5. Multiple partners with <100% condom use (referred to as “multiple partners, unprotected”).

Potential confounding factors included fixed variables collected at enrollment (i.e., workplace, charge for sex, duration of sex work, age, education level, religion, alcohol use, and douching practices) and time-dependent covariates collected at each visit (i.e., year, pregnancy status, depot medroxy-progesterone acetate use, vaginitis, cervicitis, herpes simplex type 2 serostatus, and an indicator for whether a visit occurred after a gap in attendance of more than 60 days). Year was categorized into five 3-year intervals spanning the 15-year study period. Vaginitis was defined as having trichomoniasis, bacterial vaginosis, or candidiasis.

### Statistical analysis

We first used Cox regression modeling to examine the effect of sexual risk behavior on HIV-1 acquisition. In these analyses, LTF was treated in the same way as administrative censoring. This assumption implied that LTF was independent of HIV-1 acquisition risk; the implicit assumption is that the probability of HIV-1 acquisition among women who are censored is equal to the probability of HIV-1 acquisition among women remaining in follow-up. Kaplan-Meier estimates were used to graph the probability of HIV-1 acquisition. Cox proportional hazards regression analysis was used to estimate cause-specific hazard ratios for each predictor variable. Variables included in multivariable modeling were selected *a priori* because of known or postulated associations with HIV-1 acquisition. Log-log plots of survival and Schoenfeld residuals were examined to ensure that the proportional hazards assumption was not violated.

Next, we used competing risks regression to analyze the effect of sexual risk behavior first on LTF, treating HIV-1 acquisition as a competing risk, and then on HIV-1 acquisition, treating LTF as a competing risk. A competing risks model assumes that each participant enters an initial state at the time origin, and then either remains in this state through the censoring date or enters a failure state [13]. In our case, the initial state was enrollment while HIV-1 seronegative and the two failure states were HIV-1 acquisition and LTF. A competing risk model considers the time and type of the first event only. Thus, it does not necessarily postulate, for example, that HIV-1 acquisition will not occur after LTF. In addition, competing risks regression makes no assumptions about the independence of outcomes. Specifically in this analysis, it does not make the assumption that LTF and HIV-1 acquisition are independent events. Sub-distribution hazards estimated by competing risks regression compare the probability of failure for each predictor value, conditional upon survival or occurrence of the competing risk [13].

Data analysis was performed using Stata version 11.1 (StataCorp, College Station, Texas).

## Results

### HIV-1 acquisition risk

**Figure 1:**
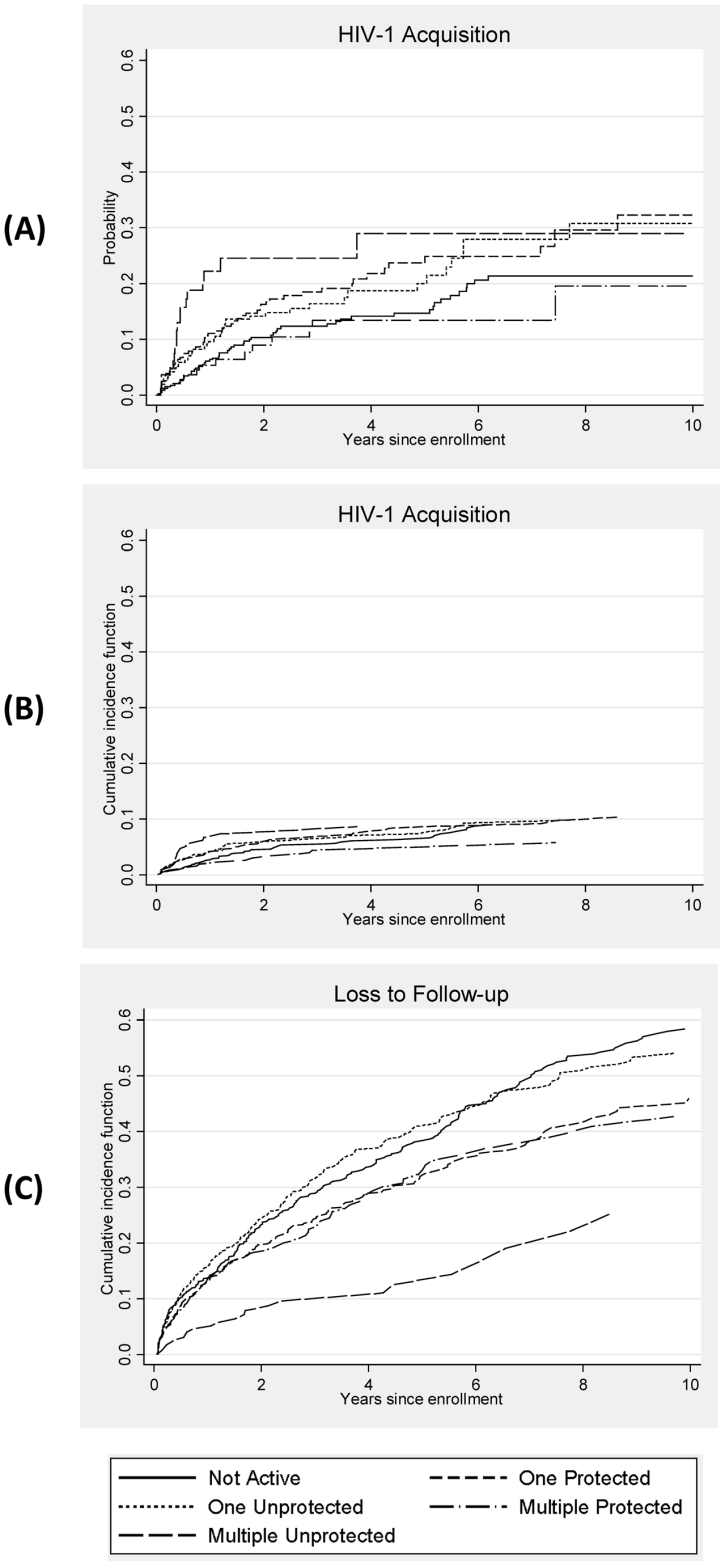
Kaplan-Meier Failure Curve and Cumulative Incidence Functions by Time-Dependent Risk Behavior Category. Panel A: Kaplan-Meier failure curve for HIV acquisition, Panel B: Cumulative incidence function for HIV acquisition, and Panel C: Cumulative incidence function for loss to follow-up. In each panel, the x axis is the probability of HIV-1 acquisition and on the y axis is years since cohort enrollment. The scale is identical in all three figures. Time-dependent sexual risk behavior is divided into 5 categories based on reported behavior in the past week: not active, protected sex with one partner (“one protected”), unprotected sex with one partner (“one unprotected”), protected sex with multiple partners (“multiple protected”), and unprotected sex with multiple partners (“multiple unprotected”), as indicated in the legend below.

Table 1 presents the baseline characteristics of the 1,513 HIV-1-seronegative women who enrolled and had follow-up HIV-1 testing. Of these women, 198 (13.1%) acquired HIV-1 infection over 4,150 person-years at risk, for an incidence of 4.8 cases per 100 person-years (py, 95% confidence interval [CI], 4.2–5.5). The median follow-up time was 16.2 months, ranging from 0.4 to 176.8 months. The median interval between the pre-infection visit and the estimated date of infection was 49 days (interquartile range [IQR], 18–140 days). A Kaplan-Meier failure curve for HIV-1 acquisition for each time-dependent risk behavior category is presented in Figure 1A. Risk category was significantly associated with HIV-1 acquisition (log-rank test p = 0.002), with the lowest HIV-free survival probability among women reporting unprotected sex with multiple partners.

**Table 1:** Enrollment Characteristics of 1,513 HIV-Seronegative Women.

*Variable* *	*N (%)*
Risk Behavior Category	
No Partner	402 (26.7)
One Partner, Protected	411 (27.3)
One Partner, Unprotected	379 (25.1)
Multiple Partners, Protected	236 (15.7)
Multiple Partners, Unprotected	79 (5.2)
Year category	
1993–1995	487 (32.2)
1996–1998	409 (27.0)
1999–2001	187 (12.4)
2002–2004	237 (15.7)
2005–2007	193 (12.8)
Workplace	
Bar or guesthouse	1106 (73.1)
Nightclub	336 (22.2)
Home-based or other	71 (4.7)
Charge category	
Non-monetary exchange	602 (39.9)
Low charge	570 (37.8)
Medium charge	138 (9.2)
High charge	197 (13.1)
Duration of sex work at enrollment	
≤1 year	852 (56.6)
>1–<4 years	315 (20.9)
≥4 years	338 (22.5)
Age at enrollment	
<23 years	361 (23.9)
23–26 years	383 (25.3)
27–30 years	340 (22.5)
>30 years	428 (28.3)
Education category	
Less than primary school	361 (23.9)
Completed primary school	599 (39.6)
Secondary school or higher	552 (36.5)
Muslim religion	191 (12.6)
Alcohol use	1169 (77.3)
DMPA use	328 (21.7)
Pregnancy	35 (2.3)
Cervicitis	210 (14.0)
Vaginitis	754 (50.1)
Herpes simplex virus type 2	1226 (81.7)
Douching behavior	
No douching	83 (5.5)
Douching with water	347 (22.9)
Douching with soap	1083 (71.6)

* N.B.: The number of observations with missing data was at most 18 (1.2%).

Time to HIV-1 acquisition was shortest for women reporting unprotected sex with multiple partners and was slightly increased for women reporting protected sex with one partner (Table 2). Compared to women with no partners, the risk of HIV-1 acquisition was not increased for women reporting unprotected sex with one partner or protected sex with multiple partners. All calendar year categories after 1995 were associated with a reduction in the hazard of HIV-1 acquisition relative to the reference category (1993–1995). Additional factors associated with reduced hazards for HIV-1 acquisition included work at a night club (versus a bar or guest house) and a preceding gap in clinic attendance >60 days. Factors associated with increased HIV-1 acquisition risk included higher educational level, vaginal douching with water or soap, and use of depot medroxy-progesterone acetate.

**Table 2:** Cox Proportional Hazards and Competing Risks Regression Results.

	Cox Regression		Competing Risks Regression		Competing Risks Regression	
	Time to HIV-1 Acquisition		Time to HIV-1 Acquisition		Time to Loss to Follow-up	
*Variable*	*aHR (95% CI)*	*P value*	*aSHR (95% CI)*	*P value*	*aSHR (95% CI)*	*P value*
Risk Behavior Category*						
No Partner	Reference		Reference		Reference	
One Partner, Protected	1.45 (0.99–2.12)	0.06	1.42 (0.96–2.11)	0.08	0.86 (0.71–1.03)	0.10
One Partner, Unprotected	1.26 (0.84–1.88)	0.26	1.05 (0.69–1.60)	0.80	1.11 (0.93–1.33)	0.26
Multiple Partners, Protected	1.29 (0.69–2.40)	0.42	1.04 (0.55–1.98)	0.89	0.77 (0.59–1.01)	0.06
Multiple Partners, Unprotected	2.41 (1.36–4.27)	0.003	2.47 (1.33–4.58)	0.004	0.50 (0.32–0.76)	0.002
Year category*						
1993–1995	Reference		Reference		Reference	
1996–1998	0.65 (0.44–0.97)	0.04	0.75 (0.50–1.13)	0.17	1.25 (0.98–1.60)	0.07
1999–2001	0.44 (0.26–0.72)	0.001	0.71 (0.44–1.14)	0.16	1.75 (1.36–2.24)	<0.001
2002–2004	0.26 (0.15–0.45)	<0.001	0.52 (0.32–0.87)	0.01	1.36 (1.05–1.75)	0.02
2005–2007	0.09 (0.04–0.20)	<0.001	0.18 (0.09–0.37)	<0.001	2.57 (2.04–3.24)	<0.001
Workplace						
Bar or guesthouse	Reference		Reference		Reference	
Nightclub	0.22 (0.12–0.42)	<0.001	0.32 (0.15–0.67)	0.002	0.94 (0.75–1.18)	0.61
Home-based or other	1.27 (0.49–3.24)	0.62	0.92 (0.35–2.40)	0.86	0.72 (0.48–1.07)	0.10
Charge category						
Non-monetary exchange	Reference		Reference		Reference	
Low charge	0.89 (0.64–1.24)	0.50	1.14 (0.82–1.59)	0.44	0.98 (0.83–1.15)	0.77
Medium charge	0.65 (0.30–1.37)	0.26	0.78 (0.35–1.76)	0.55	1.06 (0.81–1.39)	0.66
High charge	1.04 (0.53–2.04)	0.90	1.22 (0.55–2.68)	0.62	1.05 (0.80–1.37)	0.74
Duration of sex work at enrollment						
≤1 year	Reference		Reference		Reference	
>1–<4 years	0.74 (0.50–1.10)	0.13	0.92 (0.62–1.37)	0.69	0.83 (0.70–0.99)	0.04
≥4 years	0.92 (0.61–1.38)	0.68	1.21 (0.81–1.80)	0.35	0.77 (0.64–0.93)	0.007
Age at enrollment						
<23 years	Reference		Reference		Reference	
23–26 years	1.10 (0.71–1.70)	0.67	1.10 (0.70–1.72)	0.68	0.86 (0.71–1.06)	0.16
27–30 years	0.85 (0.54–1.35)	0.50	1.04 (0.64–1.69)	0.86	0.70 (0.57–0.87)	0.001
>30 years	0.67 (0.41–1.10)	0.11	0.80 (0.49–1.31)	0.37	0.71 (0.57–0.89)	0.003
Education category						
Less than primary school	Reference		Reference		Reference	
Completed primary school	1.74 (1.16–2.61)	0.007	1.47 (0.98–2.21)	0.06	0.98 (0.82–1.16)	0.79
Secondary school or higher	1.71 (1.10–2.64)	0.02	1.53 (1.01–2.31)	0.05	0.93 (0.77–1.11)	0.41
Muslim religion	1.23 (0.74–2.03)	0.42	1.12 (0.65–1.93)	0.67	0.96 (0.78–1.19)	0.70
Alcohol use	1.15 (0.79–1.67)	0.47	1.18 (0.82–1.71)	0.37	0.82 (0.70–0.96)	0.02
DMPA use*	1.62 (1.17–2.25)	0.004	1.57 (1.12–2.22)	0.01	0.78 (0.66–0.94)	0.007
Pregnancy*	1.12 (0.45–2.75)	0.81	0.72 (0.28–1.82)	0.48	2.29 (1.70–3.08)	<0.001
Cervicitis*	1.01 (0.63–1.64)	0.95	0.90 (0.53–1.53)	0.69	0.97 (0.73–1.30)	0.86
Vaginitis*	1.21 (0.90–1.62)	0.20	1.10 (0.81–1.49)	0.53	1.10 (0.96–1.26)	0.17
Herpes simplex virus type 2*	1.69 (0.94–3.03)	0.08	1.96 (1.07–3.59)	0.03	0.94 (0.78–1.14)	0.54
Douching behavior						
No douching	Reference		Reference		Reference	
Douching with water	5.90 (1.81–19.23)	0.003	3.66 (1.11–12.05)	0.03	1.18 (0.89–1.55)	0.25
Douching with soap	8.39 (2.64–26.62)	<0.001	4.41 (1.37–14.20)	0.01	1.18 (0.91–1.52)	0.20
Recent gap in attendance >60 days*	0.30 (0.22–0.42)	<0.001	0.54 (0.38–0.75)	<0.001	0.68 (0.59–0.79)	<0.001

aHR  =  adjusted hazard ratio, aSHR  =  adjusted subhazard ratio, CI  =  confidence interval, DMPA  =  depot medroxyprogesterone acetate.

* Time-dependent covariate.

### Cumulative incidence functions

**Figure 2:**
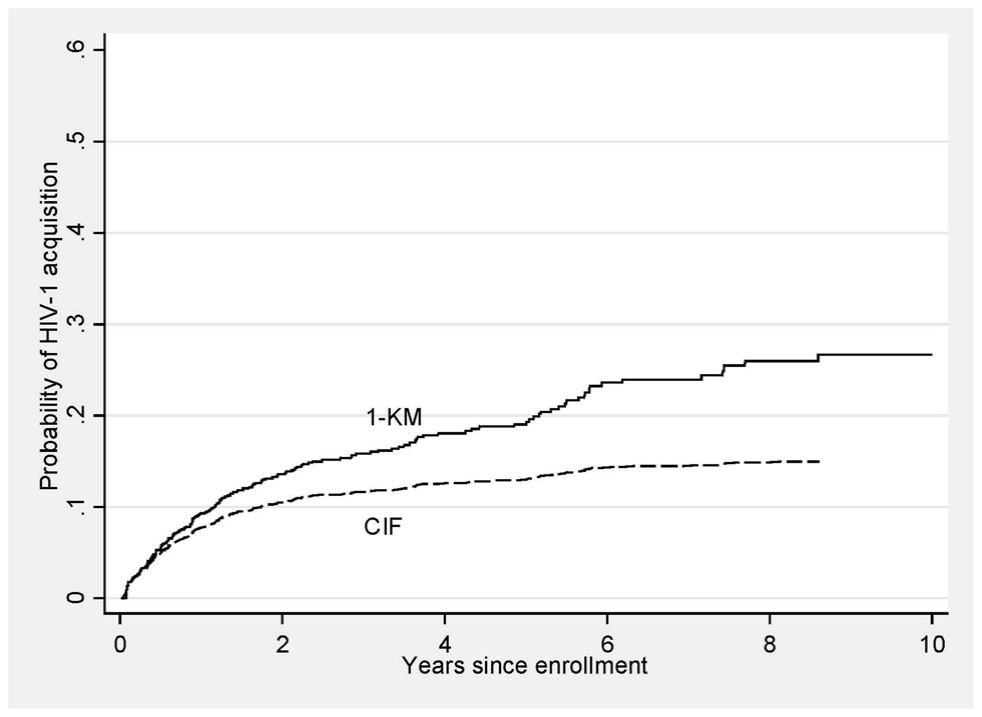
Kaplan-Meier Failure Curve and Cumulative Incidence Function for HIV Acquisition. Note that the failure curve estimates exceed the cumulative incidence function (CIF) estimates at all times during follow-up. The CIF more accurately represents the proportion of enrolled women who acquired HIV-1 infection during follow-up in the study.

Loss to follow-up can be considered a non-random competing event whose occurrence obscures the occurrence of HIV-1 acquisition for that individual. Figure 2 presents both the Kaplan-Meier failure curve (1 – KM) of HIV-1 incidence and the cumulative incidence function (CIF) based on a competing risks model. The Kaplan-Meier curve represents the probability of acquiring HIV-1 infection by each follow-up time, assuming that LTF, as well as administrative censoring, was independent of HIV-1 acquisition. In contrast, the CIF presents the probability of HIV-1 acquisition by each follow-up time, when LTF is accounted for as a competing risk. The 1 – KM curve overestimates the probability of acquiring HIV infection at each follow-up time, since individuals who were LTF were considered as censored. The 1 – KM curve will always overestimate the CIF, except in the instance when there are no competing risks, in which case the two estimates are equal. Figures 1B and 1C present the CIFs for each of the two outcomes of interest by time-dependent risk behavior category [14].

### Risk behavior and LTF

Among 1,513 women attending more than one visit, 969 (64.0%) were lost to follow-up, for an attrition rate of 23.4 per 100 py (95% CI, 21.9–24.9). Table 2 presents the adjusted results of a competing risks regression analysis of time to LTF by risk category (Competing Risks Regression, Time to Loss to Follow-up). Risk estimates in this type of regression are based on the subdistribution, or CIF for the event of interest, and are called subhazard ratios. CIFs reflect that observation of the event of interest (in this case, LTF while HIV-1-seronegative) is obscured by the occurrence of a competing risk (in this case, HIV-1 acquisition).

Women were less likely to be lost to follow-up after a visit at which they reported unprotected sex with multiple partners. Factors associated with an increased subhazard for LTF included being pregnant and calendar year category, with a shorter time to LTF in more recent years. In contrast, a reduced subhazard for LTF was associated with older age, longer duration of sex work before enrollment, alcohol use, use of depot medroxy-progesterone acetate, and a preceding gap in clinic attendance >60 days.

### Risk behavior and HIV-1 acquisition

The same competing risks regression method can be used to evaluate the risk of HIV-1 acquisition by risk behavior category, this time taking LTF into account as a competing risk. In this case, we used the CIF for HIV-1 acquisition, which reflects the fact that observation of HIV-1 acquisition during cohort follow-up can be precluded by LTF. Risk category was again significantly associated with HIV-1 acquisition (p = 0.002 in unadjusted analysis), with the lowest HIV-free survival probability among women reporting unprotected sex with multiple partners.

Table 2 presents the adjusted subhazard ratios associated with different risk behavior categories in this analysis. There was a higher risk of HIV-1 acquisition after visits on which women reported unprotected sex with multiple partners. In the adjusted model, higher education was again associated with an increased risk of HIV-1 acquisition, while factors associated with reduced risk included work at a night club (versus a bar or guest house), later calendar year (2002–2004 or 2005–2007, both compared to 1993–1995), and a preceding gap in clinic attendance >60 days. Biologic factors associated with increased HIV-1 acquisition included vaginal douching with water or soap, use of depot medroxy-progesterone acetate, and positive HSV-2 serology. Results of both analyses of HIV-1 acquisition are presented side by side for comparison.

## Discussion

Survival analysis methods are used in clinical research to describe the occurrence and timing of events that are subject to censoring and truncation [15]. When follow-up can end for several different reasons – some of which are competing risks – treating all individuals with incomplete follow-up as censored can lead to bias [16]. Competing risks regression was developed to accommodate situations in which more than one outcome is possible, observation of one outcome may obscure observation of another, and different outcomes may be correlated [13]. Although competing risks regression has been used infrequently in the HIV clinical literature, many common situations would benefit from this approach, including time to virologic suppression after treatment initiation, with stopping treatment due to toxicity as a competing risk [17], and time to AIDS-defining illness, with death as a competing risk. Using competing risks regression to analyze cohort LTF is a novel approach that at least partially addresses an important potential source of bias in observational cohort studies. Recently, a number of studies have used this approach to investigate the competing risks of LTF and mortality after HIV-infected persons initiate antiretroviral therapy (ART) [18]–[20].

Although occurrence of one outcome may obscure the occurrence of another, no assumption is made in competing risks regression about the risk of the alternate outcome or outcomes after the first event occurs [13], [21]. Competing risks regression is based on analysis of the CIF for each outcome, and reflects the probability that a given study participant will experience that specific outcome first. These functions are both intuitive and well suited to graphical display [21]. When multiple outcomes are possible, examination of the CIF for each outcome can be invaluable for analysis planning, as it provides an appreciation of each outcome's contribution to the overall fate of enrollees [22]. Comparing the CIFs for different treatment groups can also be useful in understanding how a predictor of interest is associated with each possible outcome [16].

Because individuals at risk for HIV-1 are often highly mobile, the analysis of time to HIV-1 acquisition, with LTF as a competing risk, may be of particular interest. In this study of women accepting cash or gifts in exchange for sex, risk for HIV-1 acquisition was highest when women reported unprotected sex with multiple partners. There was also an increased risk among women reporting 100% condom use with a single partner, but this estimate was relatively imprecise. Higher education level was associated with an increased hazard for HIV acquisition, as reported in several other African studies conducted during this time period [23]. As in previous cohort analyses, vaginal douching, use of depot medroxyprogesterone acetate, and positive HSV-2 serology were associated with increased HIV acquisition risk [24]–[26].

When we analyzed predictors of LTF, we found that unprotected sex with multiple partners was associated with lower attrition. Not surprisingly, pregnancy was associated with cohort attrition, while those who used depot medroxyprogesterone acetate (supplied at the clinic) were more likely to remain in follow-up. Older women and women with a longer duration of sex work were also more likely to remain, perhaps due to a sense of loyalty to the clinic and its work. It is unclear why alcohol use was associated with retention, unless this could lead to a perception of increased risk and desire for regular HIV testing.

We found that hazard ratios for HIV-1 acquisition were similar by conventional Cox regression and competing risks regression, despite differential attrition by sexual risk behavior. If both HIV-1 acquisition and LTF had been more likely among women reporting unprotected sex with multiple partners, results using the two analytic methods may have differed more dramatically, with results depending on the relative probability of each outcome. Perhaps because unprotected sex with multiple partners increased the probability of HIV-1 acquisition but decreased the probability of LTF, attrition did not substantially bias the results of Cox regression.

Loss to follow-up can create significant bias in observational studies. At least one prior study has demonstrated that retention rates among HIV-uninfected African women differ by HIV risk behavior profiles [27]. Our study suggests that, although HIV-1 status after LTF is unknown, women who leave the Mombasa Cohort may be at lower HIV risk compared to women retained in follow-up. Because loss to follow-up was, therefore, negatively correlated with event time, censoring these individuals when they leave the study overestimates the probability of experiencing HIV-1 acquisition. Studies following patients after ART initiation have used sampling-based approaches to assess the vital status of participants who were LTF [28], [29], providing the basis for a meta-analysis that has been used to obtain mortality estimates with correction for LTF [30], [31]. To our knowledge, such an approach has not been applied to HIV-seronegative individuals lost to follow-up from HIV prevention trials. Our results suggest there may be a negative correlation between HIV-1 acquisition risk and LTF, in contrast to the positive correlation between mortality and LTF in African settings [18]–[20], [30], [31]. Regardless, if an exposure of interest is a significant predictor of both the outcome of interest and LTF, analysis of both outcomes is needed for a full understanding of the data.

Overall, our results reinforce findings that individual HIV risk is highest among women who report multiple partners and unprotected sex. The slightly increased risk of HIV-1 among women reporting 100% condom use with a single partner is of unclear importance, and needs confirmation in other FSW populations. Of note, use of condoms may indicate a period of “negotiated safety” in a relationship of shorter duration or greater perceived risk [32]. Additional research is needed to confirm the hypothesis that women who report consistent condom use with a single partner are at higher risk for HIV-1 acquisition.

Our study has limitations. First, data on sexual behavior was based on self-report, which is subject to recall and social desirability biases, and often contradicts biomarker data confirming recent semen exposure, when such data are available [33]. However, sexual risk behavior data for this cohort have been correlated with biologic outcomes in this and a number of other published studies [9], [34], [35]. Second, sexual behavior was reported for the week prior to a visit, and does not include an assessment of what happened between the last visit and the beginning of the 7-day recall period. Third, “transactional” sex is a term that encompasses many types of behavior, from multiple, short-term partnerships, to a mix of short-term clients and “regular” partners, to serial relationships without overlap. We had no information on the identity of sexual partners, and so were unable to distinguish whether a woman consistently reporting one partner at visits had a single “regular” partner or a series of partners. In addition, there was no information on partner HIV-1 status or concurrency, which may confound the relationship between sexual risk behavior and outcomes. Finally, participants in this ongoing high-risk cohort, while representing a diverse group of women working in different settings and reporting different sexual behaviors, may not be representative of all risk groups. In particular, these women may be more likely to have received HIV/STI prevention information and be interested in STI screening.

In conclusion, competing risks regression is a valuable method for evaluating and interpreting time to event data when multiple outcomes are possible. Because standard survival analysis makes the assumption that outcomes are independent, it can lead to biased estimates when outcomes are correlated [16], [22]. In our analysis incorporating LTF as a competing risk, unprotected sex with multiple partners was associated with lower attrition. However, despite differential attrition by sexual risk behavior, hazard ratios for HIV-1 acquisition were similar by conventional Cox regression and competing risks regression. If LTF had been positively correlated with HIV-1 risk behavior, results using the two analytic methods may have differed more dramatically. In general, when more than one outcome is possible and observation of one outcome may obscure observation of another, competing risks regression provides a deeper understanding of the risk of each event and how different levels of a predictor of interest are related to outcomes.
